# The Disparities Researchers Equalizing Access for Minorities (DREAM) Scholars program: career development for underrepresented health equity researchers

**DOI:** 10.1017/cts.2021.845

**Published:** 2021-09-06

**Authors:** Lovoria B. Williams, Hilary L. Surratt, Victoria L. King, Philip A. Kern

**Affiliations:** 1 Center for Clinical and Translation Science, College of Nursing, University of Kentucky, Lexington, KY, USA; 2 Department of Behavioral Science, Center for Clinical and Translational Science, College of Medicine, University of Kentucky, Lexington, KY, USA; 3 Center for Clinical and Translational Science, College of Medicine, University of Kentucky, Lexington, KY, USA

**Keywords:** Career development, underrepresented faculty, mentoring, health equity

## Abstract

Diversity remains low among US colleges faculty, with only 3% identifying as Black or Hispanic. Moreover, underrepresented racial minority faculty often face unique challenges and are less likely than their white counterparts to earn higher academic rank, tenure, and funding, especially those who study health equity. We developed a novel program for health-equity focused pre-docs and junior faculty. The Disparities Researchers Equalizing Access for Minorities (DREAM) Scholars is a 24-month career development program led by the Center for Clinical and Translational Science (CCTS) that provides pilot and travel funding, career development seminars, mentoring, and writing retreats. We report the outcomes of the first Scholar cohort (N = 10), pre-docs n = 6; assistant professors, n = 4; seven were Black, one Hispanic, two White, one who identified as non-binary. At the end of the program, Scholars coauthored 34 manuscripts, 9 abstracts and 8 grants. Semi-structured interviews revealed seven major program strengths: funding, support and sense of community, accountability, exposure to translational science, network expansion, and exposure to multidisciplinary peers. Scholars provided feedback useful for subsequent cohorts. The DREAM program provided accountability and fostered a sense of community, expanded professional networks and enhanced scholarly productivity. The program serves as a model for implementation throughout the CCTSs.

## Introduction

Although the United States (US) population is becoming increasingly diverse, with 28% of the population identifying as non-White [1] diversity among US college and university faculty is lacking. According to the National Center for Education Statistics among full-time US faculty, 3% each were Black or Hispanic males or females [2]. The National Institutes of Health (NIH) has prioritized efforts to increase diversity of biomedical researchers through the support of training, development programs, and diversity supplements, yet despite these efforts, faculty diversity remains low.

Even when underrepresented racial/ethnic minority (URM) scientists attain faculty appointments, they often face unique challenges, which results in their holding lower academic rank and having higher attrition [3–5]. Moreover, the NIH reports that black researchers were significantly less likely (10.7%) than white (17.7%) to receive NIH R01 awards [6,7] partly due to their health disparities focus [7]. Moreover, URM scientists are about half as likely to receive an NIH K career development award [8]. The challenges faced by URM scientists include lack of mentoring, marginalization, feelings of isolation, overt and covert racism, and a disproportionate share of service activities that limit scholarly productivity, e.g., committee service; advisement of minority students; community service [4,6,9,10].

Evidence suggests that the barriers faced by URM faculty begin early in their academic career as pre-docs and a substantial amount of overlap exists between the academic experience of URM students and that of URM faculty [4,11]. Given the challenges known to impact URM pre-docs and junior faculty and the extant literature regarding the merits of mentoring [9,12–15], we developed an innovative career development program to support and retain health equity-focused pre-docs, post-docs, and junior faculty. This article provides a description of the program and reports initial outcomes of the Disparities Research Equalizing Access for Minorities (DREAM) Scholars Program implemented at the University of Kentucky Center for Clinical and Translational Science (CCTS).

## Methods

The purpose of the DREAM Scholars program is to promote the career development of multidisciplinary pre-docs, post-docs, and junior faculty who are interested in conducting health equity research among special populations. Because of our desire to retain URM pre-docs as future faculty, we developed the DREAM Scholars program to support pre-docs, post-docs, and junior faculty. The DREAM Scholars originally started in the College of Nursing (CON) and served as an informal mentoring program for URM individuals. In 2018, the CCTS collaborated with the CON and Center for Health Equity Transformation to expand DREAM into a structured, multidisciplinary career development program. This article describes the processes used for cohort as one of the restructured DREAM Scholars, which occurred between September 2018 and May 2020.

### Program eligibility

Applications from all individuals are accepted; however, to increase the institution-wide diversity of health equity scientists, we prioritized applicants who were URM in research. We used the NIH definition of URM, e.g. African American/Black, Hispanic American, Native American/Alaska Natives who maintain tribal affiliation or community attachment, Hawaiian Natives and natives of the US Pacific Islands; additionally, to align with the National Center for Advancing Translational Sciences definition of Integrated Special Populations (ISP), we include sexual/gender minorities in our underrepresented definition.

### Application period

We open the application period for 6 weeks and advertise the program on the CCTS webpage, list serves, and directly to campus-wide programs. Scholars are accepted after the submission of a competitive application packet that includes: (1) demographic data; (2) a letter of support from a primary mentor; (3) research plan focused on special populations; (4) career goals and objectives; (5) career development plan; and (6) applicant and mentor’s curriculum vitae. We select five applicants who display the highest potential for success as DREAM Scholars and five applicants with meritorious applications as DREAM Scholar Associates. Table 1 outlines the acceptance criteria.

**Table 1.** Scholar acceptance criteria

Underrepresented background
Evidence of interest in health equity research
Academic qualifications
Previous training performance record
History of scholarly productivity
Evidence of leadership capacity
Evidence of primary mentor’s successful mentoring

### Program leadership

The DREAM program is directed by a CCTS Associate Director who also serves as the codirector of ISP. She is an NIH-funded health equity researcher from the CON. The CCTS Career Development Director supports the program. Additionally, during the Scholar application period, one of the applicants who was a part of the original CON Scholars received a notice of award for an NIH-K01, as such; she served as a peer mentor.

### Program components

**Fig. 1.**
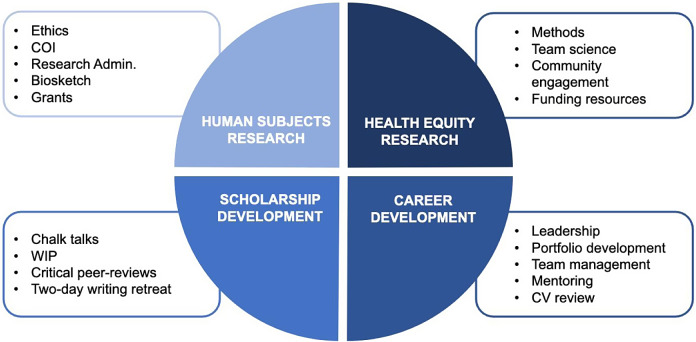
Program components.

Scholars met twice monthly for individual presentations and career development seminars. Each meeting lasted 1–1.5 hours and was held during the lunch hour; we suspended meetings during the summer. To ensure that the seminars addressed the unique needs of the Scholars, at the first meeting, we solicited the Scholars input regarding topics of interest. During the individual seminars, Scholars presented works in progress, e.g. dissertations, grants, abstracts and developed individual development plans. Faculty and staff from across campus presented the career development seminars. We structured the seminar topics into four major components as outlined in Fig. 1. Given the unique challenges faced by URM faculty and pre-docs, the career development seminars included topics geared for URMs scientists, such as enhancing negotiation skills and overcoming imposter syndrome. The program includes an annual 2-day writing retreat, which occurred at the end of each spring semester. During the writing retreat, we invited a senior scientist external to the program to meet with the junior faculty for an intense one on one review of their individual development plan.

Over the 2-year program, the Scholars received $5000 in pilot funding and $3000 travel support. Scholar Associates (hereafter included in the discussion of Scholars) participate with all DREAM activities; however, they do not receive funding support.

### Evaluation methods

We utilized a mixed methods approach to evaluate the DREAM Scholars program. The main outcome metric for the program was scholarly productivity, measured by peer-reviewed publications, and other high quality scientific outcomes, including extramural grant submissions. Publication data were obtained from Scopus for the cohort during the 2-year period of program participation, and extramural grant data from the Office of Sponsored Programs. Additionally, near the end of the cohort’s term, we sought qualitative feedback from each Scholar to examine their experiences in the program and to identify opportunities to improve the program for subsequent cohorts. Firstly, DREAM leadership met with the CCTS Director of Evaluation to discuss the program components. We then developed a semi-structured interview guide that addressed all program aspects. The Director of Evaluation attended a Scholar meeting to meet the Scholars, to establish rapport, and to inform them of her purpose for contacting them. Subsequently, she contacted the Scholars to arrange individual 1-hour semi-structured confidential interviews. To allow the Scholars the freedom to speak candidly regarding the program, none of the DREAM Scholar leaders attended the interviews. The University {blind} Office of Research Integrity approved the qualitative research procedures. Following informed consent, the Director conducted the interviews in private spaces. All interviews were audio-recorded for transcription purposes and later transcribed and verified for accuracy by evaluation staff. The CCTS evaluation team used an iterative process to identify primary themes.

## Results

The cohort included 10 Scholars, 6 were pre-docs, and 4 were assistant professors. No applications were received from post-docs. Seven were Black, one Hispanic, one White, and one who identified as non-binary. The Scholars were multidisciplinary and represented seven colleges. At the end of the 2-year program, all of the Scholars were retained. All of the Scholars had at least one peer-reviewed publication, and had collectively coauthored 34 manuscripts and 9 abstracts, obtained 8 extramural grants and acceptance into competitive national training programs. Moreover, they were recipients of numerous prestigious awards/honors.

Seven of the 10 Scholars participated in the semi-structured interview. As outlined by Table 2, seven major program strengths emerged from the semi-structured interviews.

**Table 2.** Program strengths

	Number of scholars that mentioned
1. Availability and flexibility of professional development funds	7
2. Exposure to a multidisciplinary scholar cohort	6
3. Support network and community	6
4. Network external to the home department	5
5. Exposure to diverse career stage Scholar cohort	5
6. Accountability aspects of the program	3
7. Exposure to clinical research and translational science	3

### Availability and flexibility of program funds

One Scholar stated:

*“The majority of the people in the program coming from underrepresented, you know, backgrounds, it is, I think critical to be able to provide like solid, um, funding opportunities. Because we might not always have those kinds of resources otherwise. And so I think that was honestly one of the best aspects of the program. I got to go to a very expensive conference that, you know, was super pertinent to like my research, but I would not have been able to afford to go.”*

The funding facilitated exposure to additional opportunities:

*“…the funding to be able to get professional development opportunities and go to conferences has been amazing. Because then at those places I'm able to continue getting even more like access to resources and meeting people does like continue perpetuating, the, you know, professional development goals.”*

### Exposure to a multidisciplinary Scholar cohort

One Scholar stated:

*“…. But there’s just something about, you know, us kind of all working together for our common goals and then bringing different perspectives. It’s kinda like iron sharpens iron… it could happen, you know, in a different direction if you have… homogeneous groups. But there are always gonna be more blind spots with that group in my opinion than, you know, folks who have different lenses.”*

Another stated:

“…*there’s even more diversity within the group at different perspectives. And I think that seeing things done similarly but differently is really important because as I said, when you're stuck in your little silo of your college, things are typically done a certain way and you learn that way. So it’s nice to see that you can get to the same outcome.”*

Another:

“*…it’s been great to be able to, like I said, get perspective from people who are interested in some of the same areas but who come at it from a completely different lens that’s been super informative ….”*

### Support network and community

Having a space with other URM scientists was a major theme that was mentioned by six of the Scholars. One Scholar stated:

*“… you also end up like meeting other researchers that also are minorities themselves, which is nice… it was great to go to a room and not be the only person of color or being part of the minority I guess…So I thought that’s something like unconsciously you, you've been always a minority. You kind of feel either you have to be the different person to other minority groups and talk for everyone or unconscious about it and not want to talk or seem more insecure…."*

*“When I would talk about my research, when I made the decision to focus specifically on low income African Americans…people would tell me, well, why are you focusing on black people? … Why aren't you focusing on Appalachia?… So DREAM scholars provides me a space where I don't have to constantly justify (that)…”*

### Network external to home department

The Scholars viewed the program as an opportunity to expand professional networks among the Scholars and more broadly. One Scholar commented:

*“So I'm able to get outside of my department…. it’s great to have broader perspectives… (DREAM) connects you with other folks because they have networks that they, you know, will link you with. I would say it’s extremely valuable.”*

A nurse Scholar stated:

*“…and expanding my network here at the university, um, and the resources that are available to me becoming more aware of those… I could see myself working with (my peers) in the future. We have similar research interests. We're in the same vein, but they might be looking at it from a sociological point of view and I'm looking at it from a nursing point of view.”*

### Exposure to diverse career stage

The pre-doc Scholars viewed being with Scholars of different stages as an opportunity to obtain peer mentoring and gain perspective for future roles. Scholars stated:

*“That’s a huge benefit, because even if it’s not relevant to you at this level, it’s probably going to be relevant to you in the future. So in a way you're kind of getting a glimpse of the future.”*

*“…when I was in the process of like designing the study, I was able to meet with some of the junior faculty who were able to like give me lots of advice and guidance when thinking about developing a pilot study and kind of given me their perspective from their respective fields, which I feel like was helpful.”*

### Accountability aspects of the program

Scholars viewed the program as instrumental in increasing their accountability for scholarly development.

*“I like how it’s an external structure… having milestones that you're checked up on outside of your own department, it’s like that second person.”*

*“I think having people there to review your work and telling people that I'm hoping to graduate here, I'm hoping to defend my oral qualifying exam on this day and that accountability that comes with vocalizing this to your peers and to other faculty.”*

### Exposure to clinical research and translational science

Scholars indicated that the program provided knowledge of translational science that they otherwise would not have received. One Scholar stated:

*“I'm thinking more about like translational a lot more than I did before. So we've had a lot of like presentations and didactics about like translational science and like the process of getting, you know, your science into actual practice, um, and like communicating that to other fields. I think that that’s something that has definitely sparked to me starting to think about the way that I want to approach my research more so than I had done before doing the DREAM Scholars.”*

Another stated:

*“I think that this program more than anything else I've done on campus has probably exposed me to, uh, one like the translational side of science…being in this group has allowed me to see, you know, the clinical side of things. And I think that being in this environment has exposed me to that in ways that I probably wouldn't have been exposed to…”*

The Scholars offered helpful suggestions for strengthening future cohorts as well, see Table 3. Comments ranged from expanding recruitment platforms, increasing the number of interactive sessions, restructuring the seminars to provide more opportunities to practice prior to presentations, and increasing efforts to diversify the university. Scholars suggested that appropriate places to recruit subsequent Scholars such as list serves, direct contact with associate deans for research, and doctoral program directors.

**Table 3.** Suggestions for future cohorts

	Number of scholars that mentioned
1. Expand Scholar recruitment	6
2. Increase mentoring	4
3. Restructure seminars	3
4. Use individual development plans	3
5. Increase flexibility of Scholar presentations	2
6. Expand institutional faculty and research diversity initiatives	2

### Increase mentoring

The Scholars suggested expanding the mentoring to include peer mentoring and strengthening the mentorship with their primary mentors. For instance, regarding peer mentoring one Scholar commented:

*“I think maybe have more formalized collaboration or mentorship process. It'd be nice… I think it would be cool to formalize like maybe pairing up a pre doc with one of the junior faculty or a postdoc and like working together on a project or like on a manuscript or something. I mean, that would be a cool aspect.”*

Although the Scholars were required to have a primary mentor throughout the program, many of them indicated that the mentor was not as involved as they would have liked. One Scholar stated:

*“So part of the process of applying (DREAM) is you have to identify someone to be your mentor, which is great, but then I've not seen any indication that there are checks and balances to make sure that your mentor is giving them mentorship. But I think as a formal process, I would like them to do more checks and balances probably because I've, my mentor relationship just exists on paper.”*

### Use individual development plans

The Scholars indicated a desire for having an individual development plan at the outset of the program. One Scholar stated:

*“I think, yeah, the structure and timing could be, definitely be modified. …earlier this year with someone who had sent out an IDP*, [*individual development plan]… She uses this in other contexts, and I was like, Oh, you know, for the next iteration of this, this is something that people should have at the beginning.”*

### Increase campus-wide diversity initiatives

The Scholars spoke of the need to increase racial/ethnic diversity among faculty and within the research portfolio. One Scholar stated:

*“… the experience with mentors that I've had here, outside of the DREAM program, have not been positive at all… I don't think people here, a lot of people, are comfortable even just, they're not comfortable talking about race, but just interacting with someone who’s different from them.”*

The Scholars spoke of barriers to expanding research efforts beyond Appalachia to reach racial/ethnic minority communities. One Scholar stated:

*“I submitted a letter of intent… and my work is primarily with African-Americans. Um, so yeah, it was quite discouraging to get feedback … asking for connections to a community that I had never referenced, um, in my letter of intent and so that kind of messages, um, frustrate me as a researcher. …. One of the things I didn't realize coming here was the limited research that was being done with African Americans, um, within the UK sort of research complex.”*

## Discussion

The first cohort of the DREAM Scholar program resulted in high retention and increased scholarship among the pre-docs and junior faculty members. The feedback provided important insight for future cohorts. High scholarly productivity among our Scholars are consistent with the findings of other structured programs [15] and is in part due to the mentoring, writing retreats, and the accountability measures of the program. Having the Director of Career Development as part of the leadership was a major program strength, as the Scholars received first-hand knowledge of training opportunities and application periods.

Our program is unique in that it includes individuals at different career stages [16]. Both the pre-docs and junior faculty viewed the diversity of disciplines and career stage as a program strength. The availability of travel funds, albeit small, fostered the expansion of the Scholar’s network. Given that lack of mentoring is a major challenge for URM faculty, network expansion is imperative to scientific development and future collaborations. The Scholars benefited from group mentoring from program leaders, however, although they all had a primary mentor, our evaluation findings indicate opportunities to develop the mentors in health equity research and in mentoring. Previous research indicates that URM scientists report higher quality mentor relationships when mentored by individuals who are trained in mentoring URMs [17]. In future cohorts, to monitor the mentor/mentee relationship and support mentor development, we will add mentor accountability measures, establish methods to integrate the mentors into the programing, and provide the mentors with resources to develop their mentoring skills.

Scholars expressed a sense of isolation and a feeling of lack of institutional value of health equity research outside of the program. This perspective speaks to the importance of developing institution-wide diversity and inclusivity efforts in concert with programs such as DREAM. In the fall of 2020, the University {blind} amplified institution-wide resources to advance health equity through the development of a research priority area focused on racial disparities and inequity across broad areas. The priority area will increase racial diversity at the University of Kentucky by strategic faculty recruitment, leveraging existing strengths to expand health equity research with special populations and forging new relationships.

The DREAM Scholar program was successful in providing a structured career development program for URMs. However, limitations are that it was developed to address the unique needs of University {blind} health equity-focused pre-docs, post-docs, and junior faculty. Although their needs are likely similar to elsewhere, the resources, research, and training infrastructure available at a given institution may affect Scholar’s needs and require adaptations to the program components. Secondly, although our evaluation included 70% of our Scholars, our findings would have been strengthened by full participation. There was no difference in the background or level of program engagement of the three Scholars who did not participate in the evaluation, therefore, we are confident that the experiences of the included Scholars is reflective of the entire cohort. Additionally, the scholarly productivity of the DREAM Scholars was high, however, given that DREAM is a unique inaugural program, we have no historical controls or similar programs to serve as comparison. A program design that included a comparison cohort or historical control would strengthen the validity of the results. Future career development program developers should consider using a research design to rigorously test the effectiveness of the program. Lastly, we assessed the short-term effects of the program; in the future, we will develop evaluation metrics to assess long-term effects. Given the success of cohort one, it is imperative that the program is sustained, thus dedicated institutional commitment is necessary. To date, the {blind} Cancer Center and the Cardiovascular Research Priority Areas have provided funds to support two additional Scholars for cohort 2, thereby allowing for seven funded Scholars.

In summary, the DREAM Scholar program not only promotes the career development of URM health equity investigators, but also optimizes their potential and promotes retention by fostering a sense of sense of community, belonging, and value for their health equity research. The program serves as a model for implementation throughout the CTSAs and may increase the applicant pool diversity for TL1 and KL2 programs.
